# Multiphase Identification Algorithm for Fall Recording Systems Using a Single Wearable Inertial Sensor

**DOI:** 10.3390/s21093302

**Published:** 2021-05-10

**Authors:** Chia-Yeh Hsieh, Hsiang-Yun Huang, Kai-Chun Liu, Chien-Pin Liu, Chia-Tai Chan, Steen Jun-Ping Hsu

**Affiliations:** 1 Department of Biomedical Engineering, National Yang Ming Chiao Tung University, Taipei 11221, Taiwan; kerrhsieh@nycu.edu.tw (C.-Y.H.); shoyhuang.y@nycu.edu.tw (H.-Y.H.); henry062439.be09@nycu.edu.tw (C.-P.L.); ctchan@nycu.edu.tw (C.-T.C.); 2 Research Center for Information Technology Innovation, Academia Sinica, Taipei 11529, Taiwan; t22302856@citi.sinica.edu.tw; 3 Department of Information Management, Minghsin University of Science and Technology, Hsinchu 30401, Taiwan

**Keywords:** multiphase identification, wearable inertial sensor, fall recording system

## Abstract

Fall-related information can help clinical professionals make diagnoses and plan fall prevention strategies. The information includes various characteristics of different fall phases, such as falling time and landing responses. To provide the information of different phases, this pilot study proposes an automatic multiphase identification algorithm for phase-aware fall recording systems. Seven young adults are recruited to perform the fall experiment. One inertial sensor is worn on the waist to collect the data of body movement, and a total of 525 trials are collected. The proposed multiphase identification algorithm combines machine learning techniques and fragment modification algorithm to identify pre-fall, free-fall, impact, resting and recovery phases in a fall process. Five machine learning techniques, including support vector machine, k-nearest neighbor (kNN), naïve Bayesian, decision tree and adaptive boosting, are applied to identify five phases. Fragment modification algorithm uses the rules to detect the fragment whose results are different from the neighbors. The proposed multiphase identification algorithm using the kNN technique achieves the best performance in 82.17% sensitivity, 85.74% precision, 73.51% Jaccard coefficient, and 90.28% accuracy. The results show that the proposed algorithm has the potential to provide automatic fine-grained fall information for clinical measurement and assessment.

## 1. Introduction

With the increase of life expectancy and the decrease of the fertility rate, the proportion of elders older than 64 years old in the total population explosively increases. Falls are one of the major problems leading to physical injuries, functional decline, increased healthcare costs, and even death for elders [1]. In the United States (2018), 27.5% of adults aged more than 65 years reported at least one fall in the past year, and a percentage of women reported at least one fall or fall-related injury has higher than did men [2]. Furthermore, the elders may develop anxiety, depression, and fear of repeated falling after fall events occur, which significantly influences the ability to live independently, social isolation, and the quality of life [3].

In recent years, advanced microelectromechanical systems (MEMS) and information and communication technology (ICT) create new opportunities for fall-related healthcare applications [4,5], including fall detection and prevention. Various sensors (e.g., inertial sensors [6], pressure or seismic sensors [7,8] and cameras [9,10]) and machine learning techniques (e.g., support vector machine (SVM), and k-nearest neighbor (kNN)) have been successfully applied to fall-related applications [5,11,12,13,14]. These works have shown that fall events can be automatically detected by the systems. Furthermore, the detailed information of different fall phases (e.g., the way to fall, the fall direction, the performed activities before the fall event, falling time, and landing responses) can assist in considering ways to plan preventive strategies and diagnostic approaches [12,15,16,17]. However, few works focus on the development of automatic fall recording systems to obtain fall information in fine-grained levels for clinical evaluation and measurement.

Typically, there are two common approaches to record and analyze fall-related information in clinical practice. The first is relying on self-report by fallers and caregivers [18,19]. But this approach has issues in misremembering, respondent interpretation, and cultural diversity [18,19]. Another approach is to install cameras in the potential faller’s house for long-term recording. If a fall accident has happened, clinical professionals can manually analyze fall-related information based on the video after fall events [16]. However, several challenges limit the usability of the manual-based fall analysis using cameras. The first one is that the manual operation and analysis of the whole fall event is time-consuming. Another one is that the manual recording suffers the issues in inter-rater bias and manual errors during the fall pattern analysis [16]. These issues might decrease the reliability of the analyzed results. Furthermore, it is a huge consumption of manpower and financial resources to install cameras in the potential faller’s house. To tackle the aforementioned challenges, there is a requirement to develop automatic fall recording systems to obtain objectively fine-grained fall information for fall information analysis. 

To support objective and reliable fine-grained fall monitoring, various analysis approaches of fall characteristics have been proposed in automatic fall recording systems to acquire fall information for planning fall prevention strategies [8,14,20]. These studies utilized inertial sensors and seismic sensors to acquire the movement information while falling. Then, machine learning techniques are applied to classify fall directions, fall types and fall positions. In fact, the information of other fall phases is also essential for clinical assessment and analysis. For example, the duration of resting after hitting on the ground has far greater attendant risks in dehydration, hypothermia, and even death [21]. Fall information of different fall phases (e.g., pre-fall, falling, impact, resting, and recovery phase) is important to help clinical professionals analyzing and assessing fine-grained fall information. Therefore, automatic multiple fall phase segmentation and identification are required for objective assessment and evaluation.

The main purpose of this study is to automatically obtain five fall phases for automatic fall recording systems, including pre-fall, free-fall, impact, resting, and recovery phases. This pilot study proposes an automatic multiphase identification algorithm that combines machine learning techniques and fragment modification algorithm to identify the fall phases, while most works only focused on the analysis of a single fall phase. In addition, seven types of falls emulated in a lab-based environment are conducted to validate the proposed algorithm.

## 2. Materials and Methods

### 2.1. Background

**Figure 1:**
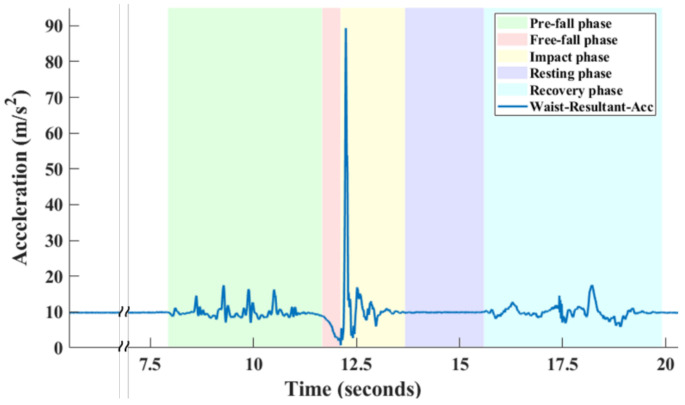
Diagram of an acceleration-based signal of a fall process.

A fall model is defined as the temporal order of the phases in a fall process. A fall process is defined as the body coming to rest unintentionally on the ground or other lower level when performing an activity, and getting up from the ground depending on consciousness [1,22]. The multiphase fall model divides a fall process into several fall phases. Various types of fall models have been proposed for the analysis of fall events [17,23,24,25,26]. There are two common types of fall models. One type is the four-phase fall model, including pre-fall, critical, post-fall and recovery phases [23]. The critical phase is the sudden body movement to the ground or other lower levels that ends with the shock. The critical phase is utilized to disclose the significant features for detecting fall events. Another work [17] proposed the five-phase fall model to obtain more detailed information of fall events for clinical analysis, which includes pre-fall, free-fall, impact, resting and recovery phases. Compared to the four-phase model, the five-phase fall model divides the critical phase into free-fall and impact phases. The information of free-fall phases can provide information of falling height and falling responses such as grabbing or stepping. In addition, the information of impact phases is important to assess fall types and directions. To provide the fine-grained fall information for clinical professionals, this study adopts the five-phase fall model. An acceleration-based signal of a fall process is shown in Figure 1. 

These phases are defined as follows:

- *Pre-fall phase (green area)*: A pre-fall phase is defined as an activity before losing balance and hitting on the ground such as walking, standing, sit-to-stand, stand-to-sit activities, that may highly impact the fall biomechanics of faller. Through identified pre-fall activities, fallers and caregivers can understand which activity easily leads to fall events.
- *Free-fall phase (red area)*: A free-fall phase is the process of sudden body movement toward the ground. There is no protective strategy that can prevent people from falling in the free-fall phase. The time of free-fall phase depends on the circumstances such as fall direction and fall height.
- *Impact phase (yellow area)*: An impact phase is the process of the person hitting on the ground and can be determined by the abrupt shock of the acceleration signal. This phase is a critical phase for fall detection algorithms and systems. The fall types and directions can be analyzed by the duration of the impact phase and the magnitude of tri-axial acceleration in the impact phase.
- *Resting phase (purple area)*: A resting phase is defined as a faller remaining inactive on the ground after a fall occurred. The injury severity of the fall affects the duration of resting phases. In some fall events, the duration of the resting phase is extremely short when the faller directly picked oneself up. Conversely, the duration of the resting phase may be long or unending if the faller is unable to rise. The situation that the faller cannot get up is identified as long-lie, which means involuntarily remaining on the ground. Then, there is no recovery phase and the fall event ends in the resting phase.
- *Recovery phase (blue area)*: A recovery phase is the last phase of a fall event if the faller has consciousness to get up from the ground. Resting and recovery phases are important to understand the severity of falls and whether the faller has immediate assistance. Previous studies [27,28] have shown the positive correlation between the mortality rates and the waiting time of rescue from falls. Rescuing the faller quickly can reduce the risks of hospitalization and death [23].

### 2.2. Developments on Multiphase Identification Algorithm

**Figure 2:**
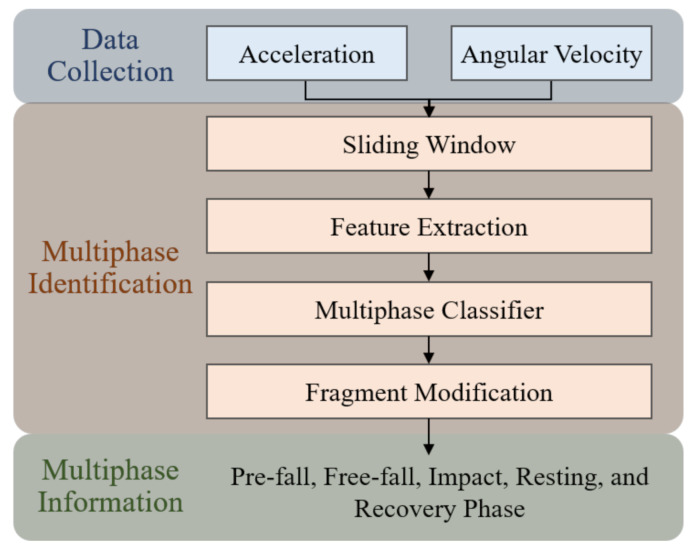
Functional diagram of proposed multiphase identification algorithm.

The functional diagram of the proposed multiphase identification algorithm is shown in Figure 2. Three main stages are included, data collection, multiphase identification, and multiphase information. Firstly, the data collected by an inertial sensor and fall-related experimental protocol are described in Section 2.2.1. Secondly, the multiphase identification utilizes machine-learning-based classifiers and fragment modification to identify fall phases, that detailed description in Section 2.2.2. Finally, the multiphase information is obtained, including the starting point, ending point and duration of each fall phase. 

#### 2.2.1. Data Collection and Experimental Protocol

Seven young adults (three males and four females; age [mean ± standard deviation]: 20.86 ± 1.07 years; height: 1.68 ± 0.08 m; weight: 64.86 ± 18.23 kg) are recruited in this study. One inertial sensor (APDM, Inc., Portland, OR, USA) is worn on the waist and utilized to collect the data of body movement. A tri-axial accelerometer, gyroscope, and magnetometer are involved in the inertial sensor. In this study, only tri-axial acceleration data from the accelerometer and tri-axial angular velocity data from the gyroscope are utilized to collect movement information, and the data are collected at a sampling rate of 128 Hz.

In the experiment, seven types and four directions of fall are executed, as presented in Table 1. One trial involves each type with one direction of fall is performed. Each type with one direction of fall repeats three times. Between trials, the resting time depends on the physical condition of the subject. In the experiment, the subjects are asked to perform the instructed fall type and get up later. For example, when performing forward fall while standing, the subjects firstly stand in front of the soft mattress, then fall forward on the soft mattress, and finally get up to stand in front of the mattress. The elapsed time of each fall type and direction is shown in Table 2. The elapsed time of each trial is defined as the duration from performing the activity before losing balance (pre-fall phase) to getting up and standing in front of the mattress (recovery phase).

**Figure 3:**
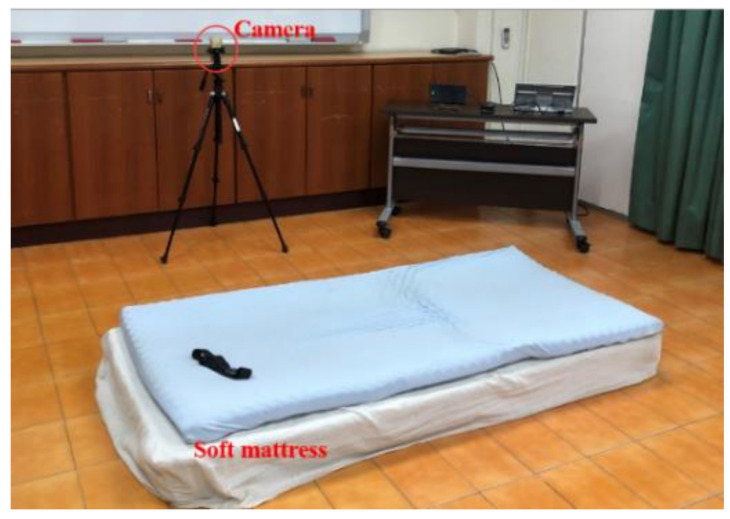
Diagram of the experimental environment setting.

**Figure 4:**
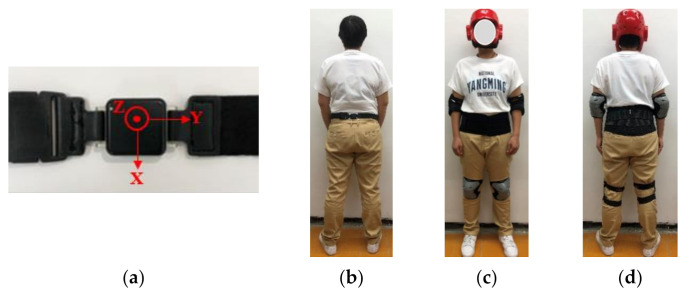
The sensor orientation, wearing position of a sensor, and the subject has worn protectors in the experiment. (**a**) Sensor orientation; (**b**) The sensor was worn on the waist (lower back); (**c**,**d**) The front and back view of the subject worn protectors, respectively.

The experimental environment setting is shown in Figure 3. All experiments were performed on the 18 cm soft mattress to prevent injuries. A helmet, a waist support belt, and knee and elbow guards are worn to protect subjects from harm in experimenting. The orientation and position of a sensor and schematic view of the subject wearing protectors are shown in Figure 4.

A camera embedded with a smartphone is synchronized with an inertial sensor to record the video with 30 frames per second and placed on the lateral side of the subjects during the entire experiment for providing ground truth labels. The researcher manually labels initial and ending timestamps of each fall phase through recorded videos. The performance of the proposed multiphase identification algorithm is evaluated by ground truth labels.

Respecting research ethics and the participant’s privacy, a required consent form was signed by the subjects, and revealing personal identities of a subject were replaced with codes. This experiment was approved by the Institutional Review Board Committee of National Yang-Ming University (YM106066E).

#### 2.2.2. Multiphase Identification

The multiphase identification includes four steps, such as sliding window, feature extraction, multiphase classifier, and fragment modification. The software MATLAB R2019a is utilized to perform and develop the collected data processing and the multiphase identification algorithm. In the sliding window, sensing data collected from an inertial sensor are divided into small segments using a window size. The influence of window size on identification performances is intelligible [29,30]. However, no clear definition exists for the selection of the optimal window size in activities identification. A large window size may involve many activities while a small window size may split an activity into several segments. Therefore, the adequate window size is needed to be investigated. In this study, five window sizes with a fixed sliding size of one sample are investigated, including window size of eight samples (0.0625 s), window size of 16 samples (0.125 s), window size of 24 samples (0.1875 s), window size of 32 samples (0.25 s), and window size of 40 samples (0.3125 s).

The segmented each segment is transformed to a set of features by the feature extraction. A total of 64 features are extracted for the multiphase identification, and that are listed in Table 3. Eight types of statistical features are extracted from each segment, including mean, standard deviation (std), variance (var), maximum (max), minimum (min), range, kurtosis, and skewness. These statistical features are commonly utilized in the field of activity recognition and identification [11,12,14]. Eight signals are utilized for features extraction, including the x-, y- and *z*-axis of acceleration ($a_{x}$, $a_{y}$ and $a_{z}$), the x-, y- and *z*-axis of angular velocity ($g_{x}$, $g_{y}$ and $g_{z}$), resultant of acceleration (*A_R_*) and resultant of angular velocity (*G_R_*). That resultant of acceleration (*A_R_*) and resultant of angular velocity (*G_R_*) are calculated by Equations (1) and (2).

(1) $$A_{R}=\sqrt{a_{x}{}^{2}+a_{y}{}^{2}+a_{z}{}^{2}}$$

(2) $$G_{R}=\sqrt{g_{x}{}^{2}+g_{y}{}^{2}+g_{z}{}^{2}}$$

The multiphase classifier uses machine learning techniques to identify each phase, including pre-fall, free-fall, impact, resting, and recovery phases. Because of the experimental protocol, the initial and ending activities are the standing activity. Therefore, the multiphase classifier may classify seven phases including the initial-static, pre-fall, free-fall, impact, resting, recovery and ending-static phases.

Five common machine learning techniques are adopted as the multiphase classifier, such as SVM, kNN, naïve Bayesian (NB), decision tree (DT), and adaptive boosting (AdaBoost). For all techniques, the training data $\left(x_{i},\;l_{i}\right),\;i=1,\;\ldots ,\;N.$ N is numbers of total training data. $x_{i}\in {\mathbb{R}}^{N}$ and the class labels are $l_{i}\in \left\{0,\;1,2,3,4,5,6\right\}$ for seven classes (0: initial-static, 1: pre-fall, 2: free-fall, 3: impact, 4: resting, 5: recovery, 6: ending-static). The introduction of these machine learning techniques and the applied parameters is as follows:

1. Support Vector Machine (SVM)

An SVM technique aims to find the optimal separating hyperplane and maximum margins in the n-dimensional feature space. The testing data are classified by the optimal hyperplane. Because the data distribution is unpredicted, there are various kernel functions for an SVM technique, such as the linear, polynomial, sigmoid, hyperbolic tangent kernel, and radial basis function (RBF). A multiclass SVM technique is implemented to classify multiphase of a fall event. In this study, the multiclass SVM classifier with the one-versus-one approach and the linear kernel function is adopted to classify multiphase of a fall event. The linear decision function is $f\left(x_{i}\right)=wx_{i}+b$, that defines the hyperplane. And, the linear kernel function $K\left(x,\;x_{i}\right)=x^{T}x_{i}$ is used for the multiclass SVM classifier.

2. K-Nearest Neighbor (kNN)

KNN is a nonparametric algorithm for recognition and classification, which stores all training data and identifies testing data are classified by a plurality vote of nearest k neighbor, which decided by the distance. There are various distance functions that can be utilized to calculate distances, such as Euclidean, Manhattan, Minkowski, Chebyshev distance functions. In this study, the Euclidean distance function $d_{i}=\sqrt{x^{2}+x_{i}{}^{2}}$ is used to calculate the distances. Because the parameter k highly depends on data distribution, it commonly uses an odd constant from 1 to $\sqrt{n}$, where n is the number of training data [31,32]. To find the best number of k for this study, a range of number between 1 and 21 with a step of 2 is explored and the best performances are gotten by k = 13.

3. Naïve Bayesian (NB)

NB technique is a conditional probability model that makes predictions using Bayes theorem with the assumption of conditional independence between all features. The posterior probability $P\left(l|x\right)=\frac{P\left(x|l\right)P\left(l\right)}{P\left(x\right)}$ for each class is calculated by prior probability $P\left(l\right)$, likelihood $P\left(x|l\right)$, and predictor prior probability $P\left(x\right)$ of class. The maximum a posterior (MAP) decision rule $\mathrm{argmax}\left(P\left(l|x\right)\right)\;$ is used to obtain the most suitable class.

4. Decision Tree (DT)

DT technique is a supervised machine learning algorithm for classification and regression. The aim is to create a model that classifies the testing data by a set of decision rules inferred from the training data. Classification and regression tree (CART), which is one of decision tree building methods, is implemented to train a multiphase classifier in this study. The splitting rule of CART is Gini impurity $I_{G}\left(x\right)=\overset{c}{\underset{l=0}{\sum }}p\left(l|x\right)\left(-p\left(l|x\right)\right)=1-\overset{c}{\underset{l=0}{\sum }}p{\left(l|x\right)}^{2}$, where c is total number of class.

5. Adaptive Boosting (AdaBoost)

Boosting methods apply a set of the weak models $h_{t}\left(x\right)$ to build a strong model $H\left(x\right)=sign\left(\overset{T}{\underset{t=1}{\sum }}\alpha_{t}h_{t}\left(x\right)\right)\;$ by a weighted vote of weak models with weight $\alpha_{t}=\frac{1}{2}\ln\frac{1-\epsilon_{t}}{\epsilon_{t}}$, where $\epsilon_{t}$ is the classification error of the weak model. AdaBoost employs decision trees as weak classifiers and a weighted sum to create a stronger classifier. Current weak classifier assigns different weighted sums to data of the previous weak classifier. The weighted sum focuses on the misclassified data of the weak classifier. The misclassified data may be assigned a higher weight to get the higher probability for classification than correctly classified data. This process is repeatedly performed until reaching the defined maximum number of iterations. The maximum number of iterations is defined as 10 in this study.

**Figure 5:**
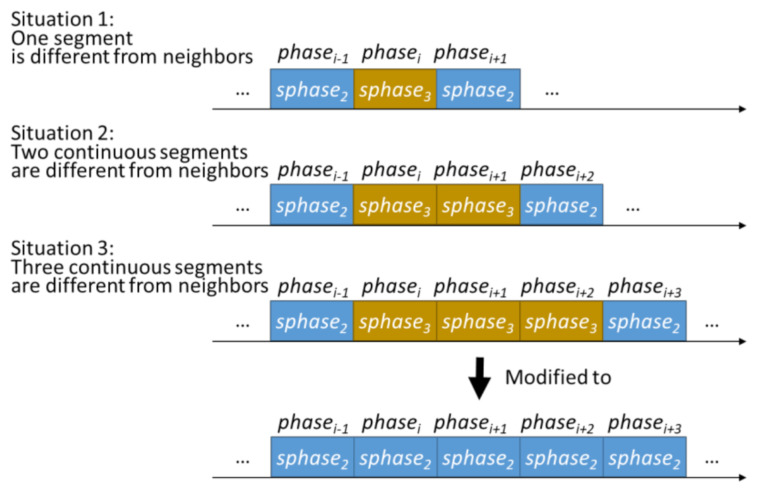
Diagram of proposed fragment modification algorithm. An example to modify one (situation 1), two (situation 2), or three (situation 3) segments that are different from previous and following segments. These segments (misclassified segments) should be modified to the same with the previous or following segments.

Finally, fragment modification is implemented to modify the misclassification results from the multiphase classification, which are an inevitable situation in machine-learning-based techniques with the sliding window approach. As shown in Figure 5, if the identified phase of one segment, or two or three segments are different from that of the previous and following segments, and the identified phase of the previous and following segments are the same phase, the one segment, or two or three segments are considered misclassified segments. The misclassified segments should be modified to the same with the previous or following segments by Algorithm 1, which is the pseudocode of the fragment modification algorithm. In Algorithm 1, each multiphase segment ($phase_{i}$) and modified multiphase segment ($mphase_{i}$) are included in $SPHASE=\left(sphase_{j}|1\leq j\leq a\right)$, where *a* is the number of defined semantic phases. There are seven defined semantic phases (*a* = 7) in this study, as following {‘initial-static’, ‘pre-fall’, ‘free-fall’, ‘impact’, ‘resting’, ‘recovery’, ‘ending-static’}. At the beginning of the fragment modification algorithm, the first segment should be set as the initial-static phase ($sphase_{1}$) and the last three segments should be set as the ending-static phase ($sphase_{7}$), referring to line 1 to line 4 and line 15 to line 18 in Algorithm 1. The main modification process refers to line5 to line 14 in Algorithm 1 to modify the one to three misclassified results between two segments with identical results.

#### 2.2.3. Multiphase Information

A segment-based sequence of the phases is obtained from the output of the fragment modification algorithm. The sample-based sequence is restored from the segment-based sequence. The multiphase information is obtained by the sample-based sequence, including starting and ending points of each phase. Furthermore, the duration of each phase can be derived from starting and ending points.
**Algorithm 1:** Fragment modification algorithm in the multiphase identification stage**Input**:An identified segments sequence $\;FALL=\left\{\left(phase_{i}|i=1,2,3,\ldots ,N\right)\right\}$, The *i*th multiphase segment $phase_{i}$; total number of segments in the sequence N
**Output**:A modified and identified segments sequence $MFALL=\left\{\left(mphase_{i}|i=1,2,3,\ldots ,N\right)\right\}$, The *i*th modified multiphase segment $mphase_{i}$
1:$phase_{1}=sphase_{1}$//$sphase_{1}$ is the semantic phase of initial-static.2:$phase_{N}=sphase_{7}$//$sphase_{7}$ is the semantic phase of ending-static.3:$phase_{N-1}=sphase_{7}$4:$phase_{N-2}=sphase_{7}$5:**for**i from 2 to N−3
**do**6: **if**
$phase_{i}$ != $phase_{i-1}$ && $phase_{i-1}$ == $phase_{i+1}$
**then**7:$phase_{i}$ = $phase_{i-1}$
8:** else if**$phase_{i}$ != $phase_{i-1}$ && $phase_{i+1}$ != $phase_{i-1}$ && $phase_{i-1}$ == $phase_{i+2}$
**then**9:$phase_{i}$ = $phase_{i-1}$
10:** else if** $phase_{i}$ != $phase_{i-1}$ && $phase_{i+1}$ != $phase_{i-1}$ && $phase_{i+2}$ != $phase_{i-1}$ && $phase_{i-1}$ == $phase_{i+3}$
**then**11:$phase_{i}$ = $phase_{i-1}$
12:  **end if**13:$mphase_{i}$ = $phase_{i}$
14:**end for**15:$mphase_{1}=sphase_{1}$16:$mphase_{N}=sphase_{7}$17:$mphase_{N-1}=sphase_{7}$18:$mphase_{N-2}=sphase_{7}$19:**return** MFALL

### 2.3. Performance Evaluation

Leave-one-subject-out cross validation (LOSOCV) is utilized to evaluate the proposed algorithm performance. It is a specific k-fold cross validation that utilizes one subject data as the testing set and the others as the training set in each fold. Therefore, 75 trials performed by the identical subjects are adopted as the testing set and 450 trials of the rest subjects are the training data for each LOSOCV round. LOSOCV iterates 7 times until each subject is used as the testing set because seven subjects are recruited in this study. 

The sample-based approach is commonly used to evaluate the reliability and performance of classification [33]. This approach calculates the number of true positive (TP), true negative (TN), false positive (FP) and false negative (FN) based on the sample-by-sample mapping between ground truth and output of multiphase identification. For example, if we want to evaluate the identification performance of the proposed algorithm on an impact phase. We define the impact phase as “positive”, and other phases as “negative”. TP is that the sample is identified as the impact phase, and the phase is performed exactly. TN is defined as that the impact phase is not performed, and the algorithm correctly predicts the sample as other phases. FP is defined as that the algorithm predicts the sample as the impact phase, but the impact phase is not performed actually. FN is that the algorithm identifies the sample as other phases, but the impact phase is performed actually. Four evaluation measures are utilized for performance evaluation, including sensitivity, precision, Jaccard coefficient, and accuracy. These evaluation measures are calculated by Equations (3)–(6). Sensitivity, precision, and Jaccard coefficient are measured for evaluation of each phase in the proposed multiphase identification algorithm. Jaccard coefficient can reveal the similarity between identified phase and the ground truth phase. In this study, Jaccard coefficient is utilized to evaluate the accuracy of locating the starting and ending points. Accuracy evaluates the average performance of the multiclass classifier:

(3) $$Sensitivity=\frac{TP}{TP+FN}$$

(4) $$Precision=\frac{TP}{TP+FP}$$

(5) $$Jaccard\;Coefficient=\frac{TP}{TP+FP+FN}$$

(6) $$Accuracy=\frac{TP+TN}{TP+FP+TN+FN}$$

**Figure 6:**
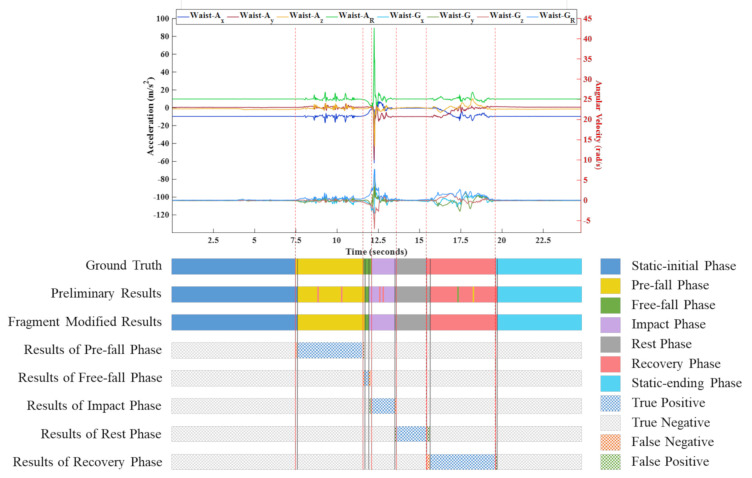
An example of a process in the multiphase identification. The fragment modified results were compared against the ground truth in terms of *TN*, *TP*, *FP* and *FN*.

An example of a process in the multiphase identification and the definition of *TP*, *TN*, *FP*, and *FN* in the sensing stream data are shown in Figure 6, which is a case of a fall event while walking.

## 3. Results

**Figure 7:**
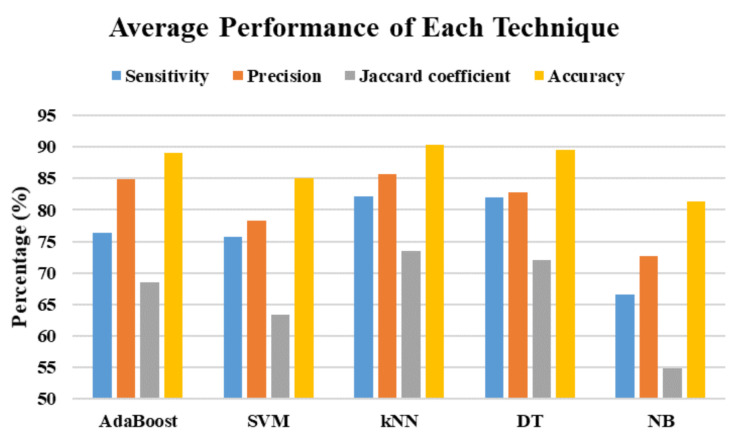
The average performance using different machine learning techniques.

**Figure 8:**
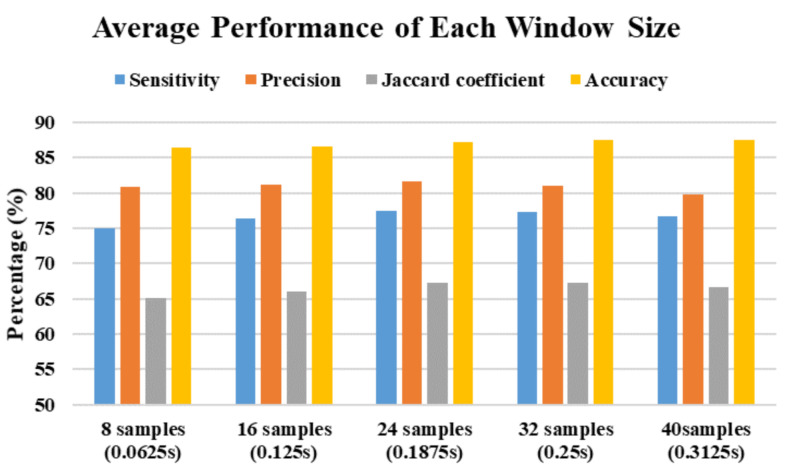
The average performance using different window sizes.

In this study, there are a total of 525 trials (75 trials × 7 subjects) are collected. LOSOCV is applied in this study, which uses 450 trials from six subjects as the training set and 75 trials from the left subject as the testing set, and iterates seven times. The average performance results of the multiphase identification algorithm using different machine learning techniques and window sizes are shown in Table 4. The overall performance of the proposed multiphase identification algorithm achieves 76.54% sensitivity, 80.89% precision, 66.45% Jaccard coefficient, and 87.05% accuracy. To summarize the machine learning techniques, the average performance is shown in Figure 7. The accuracy of all machine learning techniques with different window sizes is over 80%. The proposed algorithm using the kNN technique achieves the best performance in 82.17% sensitivity, 85.74% precision, 73.51% Jaccard coefficient, and 90.28% accuracy. Then, the proposed algorithm using the NB technique has the worst performance than that using other techniques. To summarize the window sizes, the average performance is shown in Figure 8. The best performance of the proposed algorithm with different window sizes in sensitivity, precision, Jaccard coefficient, and accuracy is 77.40% with a window size of 24 samples (0.1875 s), 81.59% with a window size of 24 samples (0.1875 s), 67.28% with a window size of 32 samples (0.25 s), and 87.52% with a window size of 40 samples (0.3125 s), respectively.

**Figure 9:**
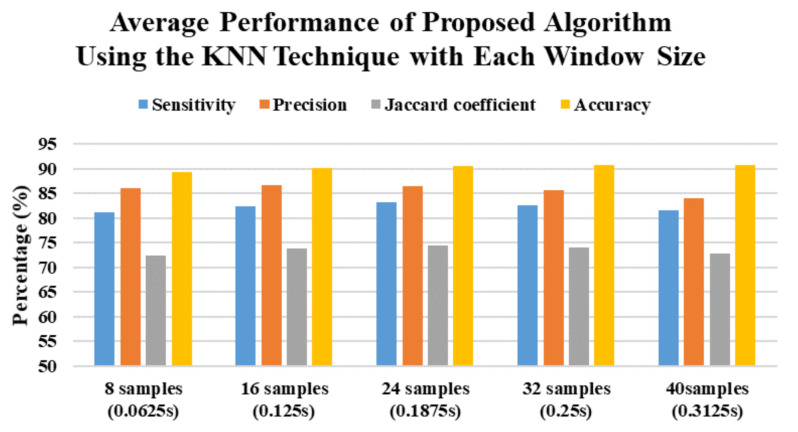
The average performance of proposed algorithm using the kNN technique with each window size.

According to the best performance of machine learning techniques, Table 5 shows the performance results of each phase using the kNN technique with different window sizes. In summary, the average performance of proposed algorithm using the kNN technique with each window size is shown in Figure 9. The highest sensitivity, precision, Jaccard coefficient and accuracy occurred proposed algorithm using the kNN technique with a window size of 24 samples (0.1875 s), 16 samples (0.125 s), 24 samples (0.1875 s) and 32 samples (0.25 s), respectively. For all window sizes, the sensitivity and Jaccard coefficient of the resting phase and the precision of the pre-fall phase outperform that of other phases, except the static phase. The sensitivity, precision, and Jaccard coefficient of the free-fall phase are worse than that of other phases.

## 4. Discussion

The proposed multiphase identification algorithm can automatically and objectively identify fall phases using a single wearable inertial sensor. The proposed algorithm combines machine learning techniques and fragment modification algorithm to provide fine-grained fall information about starting point, ending point and duration of fall phases for clinical professionals. The proposed multiphase identification algorithm using the kNN technique can achieve the best performance in all measures. The results demonstrate the kNN technique is more suitable for the proposed algorithm. Moreover, the kNN technique has the advantages of less computation complexity. The window size is an important parameter that may affect identification performance. Larger window sizes may include more patterns and characteristics across phases that obscure the machine learning techniques to build the models and lead to misidentification. Smaller window sizes may not include patterns and characteristics of a whole phase that confuses to build the models and easily lead to motion fragment by trained models. Over the five window sizes, a window size of 24, 16, 24, and 32 samples can achieve the best average performance using the kNN technique in sensitivity, precision, Jaccard coefficient, and accuracy, respectively. Therefore, the window size using 16, 24, and 32 samples are suitable for the proposed multiphase identification algorithm.

To obtained the initial and ending points of a whole fall event, the movement of initial and ending posture was collected in this experiment. Therefore, the initial- and ending-static phases are included in the proposed multiphase identification algorithm. The Jaccard coefficient is defined as the samples of the intersection divided by the samples of the union in the target phase between ground truth and identification result. The initial- and ending-static phases have the best performance in the Jaccard coefficient. The free-fall phase identification has the worst performance of the Jaccard coefficient because the free-fall phase has a short duration and be confused with daily activities easily. Therefore, the free-fall phase identification remains room for improvement.

A summary of previous studies [8,14,16,17,20] on sensor types, techniques, and provided fall-related information is shown in Table 6. Most studies focused on extracting characteristics of a whole fall to obtain information of fall directions, fall types and fall positions. Hsieh et al. [20] proposed a machine-learning-based algorithm to detect fall directions, and the accuracy was 97.34. Hussain et al. [14] used machine-learning-based classifiers to detect fall types, and the results was 96.82% accuracy using random forest classifier. Clemente et al. [8] utilized seismic sensor signals to estimate fall positions using the time difference of arrivals measurements, and results showed that the localization error is smaller than 0.28 m. However, these studies only focused on extracting characteristics using the entire fall signal, and the fine-grained information and characteristics were not extracted. 

Two common approaches collected signals by inertial sensors and cameras to manually label the starting and ending points of fall phases and obtain the fine-grained fall information. The first is self-reports by fallers and caregivers [18,19]. The detailed fall-related information, including falling time, the activities before falling and fall direction, is recorded manually. The second approach is to install cameras in the potential faller’s house for long-term recording. After fall events occur, clinical professionals can manually analyze fall-related information based on the videos [16]. However, these two approaches may suffer issues in inter-rater bias and manual errors. Especially, the camera-based approach captured only 28% and 45% of fall events in two different care facilities. To our best knowledge, this is the first study aiming to automatically and objectively identify fall phases within a fall process using machine learning techniques. Machine learning techniques have been proposed and applied to automatic identification of activity, gesture and movement in other applications [34,35,36,37]. However, obtaining fine-grained fall information still rely on manual execution [16,17]. Our study demonstrates the feasibility of multiphase identification for phase-aware fall recording system.

The performances of the proposed algorithm are restricted by some challenges to multiphase identification, such as motion variability, temporal order modification, boundary decision. The performance of parts of phases needs to be improved, such as the free-fall phase especially. We plan to examine other algorithms and powerful learning techniques, such as hierarchical algorithms with rule conditions and machine learning techniques, convolutional neural networks (CNN), long short-term memory (LSTM). Another limitation is that only seven subjects with narrow age distributed are recruited and seven types of fall are performed in the experimental environment for validation of the proposed multiphase identification algorithm. More subjects with wide age distributed, types of fall and real-world fall events datasets will be investigated to validate the proposed multiphase identification algorithm in the future.

## 5. Conclusions

To obtain and understand the phase information of fall events for clinical requirements, an automatic multiphase identification algorithm is proposed for phase-aware fall recording systems. The sliding window approach, several machine learning techniques, and fragment modification algorithm are utilized to identify five phases in a fall event. The proposed multiphase identification algorithm using the kNN technique with a window size of 24 samples could achieve the best performance in 83.09% sensitivity, 86.53% precision, 74.47% Jaccard coefficient, and 90.56% accuracy. The results show that the proposed system has the potential to provide automatic and reliable fine-grained fall information for clinical professionals.

## Figures and Tables

**Table 1:** Types and directions of fall.

No.	Type	Direction	Trial
1	Fall while standing	Forward, backward, right lateral, and left lateral	84
2	Fall while standing up	Forward, backward, right lateral, and left lateral	84
3	Fall while sitting down	Forward, backward, right lateral, and left lateral	84
4	Fall while stooping down	Forward, backward, right lateral, and left lateral	84
5	Fall while walking	Forward, backward, right lateral, and left lateral	84
6	Fall while jumping	Forward, backward, right lateral, and left lateral	84
7	Fall while walking backward	Backward	21

**Table 2:** Elapsed time of each fall type and direction (notation: mean ± standard deviation).

Type	Direction			
	Forward (s)	Backward(s)	Right Lateral(s)	Left Lateral(s)
Fall while standing	14.89 ± 2.03	14.91 ± 2.53	15.58 ± 2.32	15.35 ± 1.95
Fall while standing up	16.66 ± 3.01	17.00 ± 2.58	17.07 ± 1.83	17.33 ± 1.67
Fall while sitting down	16.44 ± 1.88	15.91 ± 1.85	15.90 ± 1.85	15.69 ± 1.72
Fall while stooping down	17.68 ± 1.35	20.56 ± 2.73	18.61 ± 2.13	19.07 ± 1.82
Fall while walking	15.52 ± 1.97	16.46 ± 1.93	15.65 ± 1.73	15.81 ± 1.55
Fall while jumping	19.04 ± 2.23	19.64 ± 2.62	19.11 ± 2.41	19.02 ± 2.36
Fall while walking backward	--	19.17 ± 1.80	--	--

**Table 3:** The feature set for multiphase classifier.

Feature Set, F = (f_1_, f_2_, …, f_64_) ϵ R_64_	Feature Description
f_1 ~_ f_8_	$mean,\;std,\;var,\;max,\;min,\;range,\;kurtosis\;and\;skewness\;of\;a_{x}$
f_9 ~_ f_16_	$mean,\;std,\;var,\;max,\;min,\;range,\;kurtosis\;and\;skewness\;of\;a_{y}$
f_17 ~_ f_24_	$mean,\;std,\;var,\;max,\;min,\;range,\;kurtosis\;and\;skewness\;of\;a_{z}$
f_25 ~_ f_32_	$mean,\;std,\;var,\;max,\;min,\;range,\;kurtosis\;and\;skewness\;of\;g_{x}$
f_33 ~_ f_40_	$mean,\;std,\;var,\;max,\;min,\;range,\;kurtosis\;and\;skewness\;of\;g_{y}$
f_41 ~_ f_48_	$mean,\;std,\;var,\;max,\;min,\;range,\;kurtosis\;and\;skewness\;of\;g_{y}$
f_49 ~_ f_52_	$mean,\;std,\;var,\;max,\;min,\;range,\;kurtosis\;and\;skewness\;of\;A_{R}$
f_53 ~_ f_64_	$mean,\;std,\;var,\;max,\;min,\;range,\;kurtosis\;and\;skewness\;of\;G_{R}$

**Table 4:** The performance results of the multiphase identification algorithm using machine learning techniques versus window sizes (unit:%).

Machine Learning Technique	Evaluation Measure	Window Size					
		8 Samples (0.0625 s)	16 Samples (0.125 s)	24 Samples (0.1875 s)	32 Samples (0.25 s)	40 Samples (0.3125 s)	Overall
AdaBoost	Sensitivity	73.25	75.07	77.31	78.06	77.87	76.31
	Precision	83.82	85.81	86.55	85.31	83.04	84.91
	Jaccard coefficient	65.23	67.35	69.73	70.26	69.83	68.48
	Accuracy	87.85	88.57	89.30	89.54	89.77	89.00
SVM	Sensitivity	72.47	74.44	75.70	77.62	78.22	75.69
	Precision	78.40	76.61	77.92	79.07	79.30	78.26
	Jaccard coefficient	61.81	61.44	63.05	64.91	65.37	63.32
	Accuracy	84.45	83.83	84.80	85.86	86.22	85.03
kNN	Sensitivity	81.17	82.46	**83.09**	82.65	81.49	**82.17**
	Precision	86.02	**86.70**	86.53	85.56	83.91	**85.74**
	Jaccard coefficient	72.33	73.83	**74.47**	74.05	72.86	**73.51**
	Accuracy	89.32	90.07	90.56	**90.76**	90.69	**90.28**
DT	Sensitivity	81.84	82.42	82.84	81.61	80.92	81.93
	Precision	83.57	83.33	83.51	82.19	81.28	82.78
	Jaccard coefficient	72.46	72.51	73.03	71.64	70.93	72.12
	Accuracy	89.75	89.56	89.86	89.36	89.26	89.56
NB	Sensitivity	65.80	67.44	68.07	66.92	64.76	66.60
	Precision	72.77	73.01	73.41	73.26	71.31	72.75
	Jaccard coefficient	53.55	54.93	55.64	55.56	54.36	54.81
	Accuracy	80.76	81.24	81.50	81.72	81.67	81.38
Overall	Sensitivity	74.91	76.37	**77.40**	77.37	76.65	**76.54**
	Precision	80.92	81.09	**81.59**	81.08	79.77	**80.89**
	Jaccard coefficient	65.08	66.01	67.19	**67.28**	66.67	**66.45**
	Accuracy	86.42	86.65	87.20	87.45	**87.52**	**87.05**

**Table 5:** The performance results of each phase using the kNN technique with different window sizes (unit:%).

	Using a kNN technique with a window size of 8 samples (0.0625 s)							
	Initial-static	Pre-fall	Free-fall	Impact	Resting	Recovery	Ending-static	Overall
Sensitivity	99.70	62.96	51.01	79.64	96.33	80.12	98.46	81.17
Precision	90.26	93.18	67.03	87.00	84.12	88.19	92.39	86.02
Jaccard coefficient	90.03	60.17	40.20	71.07	81.50	72.31	91.05	72.33
Accuracy								89.32
	Using a kNN technique with a window size of 16 samples (0.125 s)							
Sensitivity	99.70	64.34	54.42	81.21	96.74	82.38	98.45	82.46
Precision	91.38	94.75	67.63	86.49	86.07	87.68	92.88	**86.70**
Jaccard coefficient	91.14	62.11	42.73	71.94	83.62	73.79	91.51	73.83
Accuracy								90.07
	Using a kNN technique with a window size of 24 samples (0.1875 s)							
Sensitivity	99.54	66.02	54.89	82.07	96.98	83.80	98.33	**83.09**
Precision	92.50	95.73	64.50	85.12	87.31	87.52	93.03	86.53
Jaccard coefficient	92.12	64.10	42.08	71.68	84.97	74.81	91.55	**74.47**
Accuracy	--	--	--	--	--	--	--	90.56
	Using a kNN technique with a window size of 32 samples (0.25 s)							
Sensitivity	99.29	67.71	49.88	81.87	96.86	84.70	98.24	82.65
Precision	93.44	96.18	57.93	82.93	88.07	87.49	92.89	85.56
Jaccard coefficient	92.83	65.91	37.11	70.01	85.60	75.52	91.35	74.05
Accuracy	--	--	--	--	--	--	--	**90.76**
	Using a kNN technique with a window size of 40 samples (0.3125 s)							
Sensitivity	99.05	68.82	41.97	80.79	96.34	85.22	98.28	81.49
Precision	94.24	96.01	48.38	80.30	88.51	87.25	92.66	83.91
Jaccard coefficient	93.40	66.91	29.82	67.38	85.61	75.75	91.15	72.86
Accuracy	--	--	--	--	--	--	--	90.69

**Table 6:** A summary of previous studies on sensor types, techniques, and provided fall-related information.

Article (Year) [Reference]	Sensor Type	Technique (Method)	Provided Fall-Related Information
Becker et al. (2012) [17]	Inertial sensor	Manual labeling	Starting and ending points of fall phases
Robinovitch et al. (2013) [16]	Camera	Manual labeling	Causes of falling; Activities before the fall event.
Hsieh et al. (2018) [20]	Inertial sensor	Machine learning (SVM)	Fall directions (97.34% accuracy)
Hussain et al. (2019) [14]	Inertial sensor	Machine learning (kNN, SVM and random forest)	Fall types (96.82% accuracy using random forest classifier)
Clemente et al. (2019) [8]	Seismic sensor	Machine learning (SVM)	Fall positions (localization error is smaller than 0.28 m)
This study	Inertial sensor	Machine learning (SVM, kNN, NB, DT and AdaBoost)	Starting and ending points of fall phases; Duration of fall phases.

## Data Availability

The data presented in this study are available on request from the corresponding author. The data are not publicly available due to privacy.
